# Older Swedish Adults with High Self-Perceived Health Show Optimal 25-Hydroxyvitamin D Levels Whereas Vitamin D Status Is Low in Patients with High Disease Burden

**DOI:** 10.3390/nu8110717

**Published:** 2016-11-11

**Authors:** Martin Carlsson, Pär Wanby, Lars Brudin, Erik Lexne, Karin Mathold, Rebecca Nobin, Lisa Ericson, Ola Nordqvist, Göran Petersson

**Affiliations:** 1 Department of Clinical Chemistry, County Hospital of Kalmar, 391 85 Kalmar, Sweden; 2 Department of Medicine and Optometry, Linnaeus University, 392 34 Kalmar, Sweden; lisa.ericson@lnu.s (L.E.); goran.petersson@lnu.se (G.P.); 3 Section of Endocrinology, Department of Internal Medicine, County Hospital of Kalmar, 391 85 Kalmar, Sweden; par.wanby@ltkalmar.se; 4 Department Medical and Health Sciences, University of Linkoping, 581 83 Linköping, Sweden; lars.brudin@ltkalmar.se; 5 Department of Clinical Physiology, County Hospital, 391 85 Kalmar, Sweden; 6 Department of Psychiatry, County Hospital of Kalmar, 391 85 Kalmar, Sweden; erik.lexne@ltkalmar.se; 7 Department Geriatric Medicine, County Hospital of Kalmar, 391 85 Kalmar, Sweden; karin.mathold@ltkalmar.se; 8 Department of Orthopaedics, County Hospital of Kalmar, 391 85 Kalmar, Sweden; rebecca.nobin@ltkalmar.se; 9 The Pharmaceutical Department, Kalmar County Council, 392 44 Kalmar, Sweden; ola.nordqvist@ltkalmar.se

**Keywords:** vitamin D, 25-hydroxyvitamin D, Sweden, emergency care, frail older adult

## Abstract

Controversy pervades the definition of adequate and optimal vitamin D status. The Institutes of Medicine have recommended serum 25(OH)D levels above 50 nmol/L based upon evidence related to bone health, but some experts, including the Endocrine Society and International Osteoporosis Foundation, suggest a minimum serum 25(OH)D level of 75 nmol/L to reduce the risk of falls and fractures in older adults. In a cross-sectional study, we compared vitamin D status in people ≥75 years selected from four groups with a frailty phenotype, combined with a control group free from serious illness, and who considered themselves completely healthy. Only 13% of the 169 controls were vitamin D deficient (S-25(OH)D) < 50 nmol/L), in contrast with 49% of orthopedic patients with hip fractures (*n* = 133), 31% of stroke patients (*n* = 122), 39% of patients visiting the hospital’s emergency department ≥4 times a year (*n* = 81), and 75% of homebound adult residents in long-term care nursing homes (*n* = 51). The mean vitamin D concentration of the healthy control group (74 nmol/L) was similar to a suggested optimal level based on physiological data and mortality studies, and much higher than that of many officially recommended cut-off levels for vitamin D deficiency (<50 nmol/L). The present study provides a basis for planning and implementing public guidelines for the screening of vitamin D deficiency and vitamin D treatment for frail elderly patients.

## 1. Introduction

Hypovitaminosis D is common in older adults [1]. Older subjects with 25-Hydroxyvitamin D (25(OH)D) levels below 50–70 nmol/L have a significantly higher risk of earlier death than those with higher 25(OH)D levels [2,3,4]. Vitamin D deficiency may result in osteopenia/osteoporosis, muscle weakness, and atrophy (sarcopenia), poor balance, and an increased risk for fractures [5]. Aside from these well-known classical effects of vitamin D deficiency, non-classical effects of vitamin D deficiency such as insulin resistance, increased risk of infection, many forms of cancer, cardiovascular disease etc., are also associated with vitamin D inadequacy. Most of the current data regarding these non-classical effects of vitamin D deficiency are, however, based on observational and epidemiological studies, which do not prove causality between vitamin D deficiency and overall poor health.

Vitamin D deficiency is also associated with lower quality of life [6]. A recent meta-analysis showed some evidence that vitamin D3 supplementation may reduce mortality in older adults [7], as well as a recent study showed that genetically low vitamin D concentrations increase mortality [8].

The major source of vitamin D is from sensible sunlight exposure. Sweden’s geographical location with limited sunlight during the year implies that skin can produce sufficient vitamin D only during the summer months. Studies in healthy blood donors in Sweden (18–65 years of age) have shown that serum 25-hydroxy vitamin D (S-25(OH)D) levels vary greatly over the year, and correlate with the intensity of UVB irradiation [9]. Older adults usually spend less time in the sun than younger and have a lower capacity for their skin to produce vitamin D. Sweden has an aging population, and one of the highest reported incidences of hip fractures. There is also a seasonal variation for hip fracture, with the highest risk in winter, and the lowest in summer [10]. The economic burden of incidental fragility fractures in Sweden is very high [11].

Some studies have found a high prevalence of vitamin D deficiency among elderly Swedes, especially among those in institutions [3], but others have shown almost normal vitamin D status in certain groups of older people [2,12]. However, it is difficult to compare these studies, as the vitamin D status in elderly people varies widely in different studies, depending on the population studied, method for determining vitamin D status, residence, and timing of data collection.

In the latest Nordic Nutritional Recommendations 2012 (NNR5), published in 2013, the recommended daily intake of vitamin D for those over 75 years of age was raised from 10 µg (400 IU)/day to 20 µg (800 IU)/day, since a protective effect of such an intake against mortality, fractures, and falls was considered to be scientifically supported [13]. In the same document a serum-25(OH)D concentration of >50 nmol/L was used as an indicator of sufficient vitamin D status. To prevent vitamin D deficiency, (June 2015) the Swedish National Food Agency recently proposed that the number of foods requiring mandatory vitamin D fortification in Sweden should be increased.

Despite evidence of the importance of vitamin D in various health conditions, there is a lack of scientific consensus concerning optimal 25(OH)D concentrations for older people. To the best of our knowledge, there are no other Swedish studies employing novel laboratory methods estimating 25(OH)D concentrations in the very old who feel healthy. We believe that our study of such an elderly, but healthy group can provide an indication of what optimal vitamin D level health care should strive for. We also chose to study vitamin D status in ill older adults in order to aid health care in its objective to provide guidelines of screening for vitamin D deficiency and vitamin D treatment for these groups of frail elderly patients.

To answer these questions, we selected patients from different vitamin D deficiency risk populations, and compared them with a healthy age-matched control group. Patients from four high-risk populations were investigated in the study. As a control group, age-matched healthy older adults (Controls) were investigated.

Our hypotheses stated that frail older adults from the risk groups would have a suboptimal level of vitamin D, and few would be substituted with vitamin D and calcium. We also sought to investigate the vitamin D status of healthy, but very old, people.

## 2. Experimental Section

### 2.1. Study Population and Study Design

The study was conducted between February 2014 and April 2015 at Kalmar County Hospital in the southeast of Sweden (latitude 56.7° N). To investigate possible seasonal variation in vitamin D status, patients and controls were recruited evenly with regard to season.

This study was conducted according to the guidelines laid down by the Declaration of Helsinki. The Regional Ethical Review Board at Linköping University approved the study, and informed consent was obtained from all participants.

Inclusion criteria for all participants were age ≥75 years. All participants came from the same geographical area (Kalmar County, in southeastern Sweden). Subjects from the following vitamin D deficiency risk populations were consecutively entered into the study: patients admitted to the orthopedic ward following an osteoporotic hip fracture (Fracture), patients with acute stroke admitted to a stroke ward (Stroke), and patients who frequently used local emergency care with ≥4 visits per year (Frequent Emergency Users; FEU). As a fourth group we included homebound elderly residents in long-term care nursing homes (Nursing homes)). These older, multi-ill persons, living in nursing homes had poor access to outdoor activities and could thus develop vitamin D deficiency. When patients were unable to understand because of dementia or stroke, a family member able to advise on the presumed wishes of the patient was approached to act on behalf of the patient.

Healthy volunteers (Controls) ≥75 were enrolled as the control group. A variety of strategies was used for recruitment, including advertising in local newspapers, supermarkets, and pensioner associations. These older adult controls were enrolled in the study only if they considered themselves to be in good health despite their age (a high degree of physical and emotional well-being) and were satisfied with their current life-situation.

### 2.2. Measurements

All participants underwent anthropometric measurement and biochemical screening on the same occasion. Systolic and diastolic blood pressure was measured on the right arm in the supine position. The medical history was taken on inclusion, obtained through a nurse’s interview. A standard pretested questionnaire was used for recording baseline information. Skin color was recorded using the Fitzpatrick scale. For all participants, self-reported current medication data was supplemented by medication lists in electronic medical records (Cambio Cosmic).

Height and weight were measured with the subject wearing light clothing without shoes. Body mass index (BMI) was calculated as kilograms per square meter. Waist circumference was measured midway between the lowest rib and the iliac crest with the subject in the supine position.

All included subjects in the control group had rated their health as very good despite their old age.

The question of how they rated their health was asked by the nurse before the examination by a telephone interview and also during the visit. Only the older controls who rated their own health as very good/excellent were included in the study. Subjects in the healthy control group were also asked about previous medical history including history of dementia, low energy fractures, and stroke. If they had had any of these diseases, they were excluded from the study. Subjects in the control group who were prescribed antidiabetic medication, vitamin D, or calcium by a physician were also excluded.

### 2.3. Blood Samples

Venous blood samples were collected for quantification of different biochemical parameters.

For patients in the stroke and emergency department groups, non-fasting blood was drawn in the early morning (7:00–9:00 a.m.), the day after arrival to the hospital. From patients in the fracture group, investigations and blood samples were performed early in the morning (7:00–9:00 a.m.). Blood was drawn from the patients 3.8 (0–6) days after hospitalization for hip fracture and, in most cases, three days following surgery, which was performed on arrival or the following day.

Analyses were performed at the Kalmar County, Clinical Chemistry Hospital Laboratory, and all steps were performed according to the manufacturers’ recommended protocol.

Blood samples were analyzed for plasma calcium, albumin, phosphorus, Alkaline Phosphatase (ALP), and creatinine using VITROS^®^ 5,1 FS Chemistry System from Ortho Clinical Diagnostics, Rochester, NY, USA. Blood HbA1c and serum intact parathyroid hormone (PTH) were analyzed using Cobas C501, and Cobas e 601 chemistry and immunology analyzers by Roche Diagnostics, Basel, Switzerland. Serum Ionized calcium was analyzed using ABL 825 (Radiometer Medical ApS, Bronshoj, Denmark). The serum level of total 25-hydroxyvitamin D (calcidiol or 25(OH)D) is most often considered the best indicator of vitamin D status. As an indicator of vitamin D status we used total serum 25-hydroxyvitamin D (25-OH-vitamin D2 + 25-OH-vitamin D3), which was measured by tandem mass spectrometry (LC-MS/MS) using the API 4000 instrument with an atmospheric pressure chemical ionization (APCI) source (AB Sciex, Concord, ON, Canada) and UFLC Schimadzu Prominence (Schimadzu, Kyoto, Japan) [14]. The calibrators used were traceable to the National Institute of Standards and Technology (NIST), standard reference material (SRM) 972a, Vitamin D in Human Serum. The laboratory participated among other laboratories (*n* = 997, April 2014) in the Vitamin D external quality assessment scheme (DEQAS), and were within ±1 SD of the target value and method mean (LC-MS/MS) during the study period. The laboratory met the criteria for a proficiency certificate available to successful participants.

The prevalence of vitamin D deficiency depends on the cut-off point used. We used the American Endocrine Society Clinical Practice Guideline, published 2011, and defined the optimal vitamin D level as a level of 25(OH)D > 75 nmol/L. Vitamin D insufficiency was set at a 25(OH)D level between 50 and 75 nmol/L, while vitamin D deficiency was defined as a 25(OH)D level < 50 nmol/L. Highly deficient status was set at a 25(OH)D level < 25 nmol/L.

### 2.4. Statistical Methods

The results for variables are presented as the mean (SD), the median (range), and for categorical variables as counts (%). *p*-values from Mann-Whitney’s U-test are presented, provided Kruskal-Wallis nonparametric ANOVA was statistically significant (*p* < 0.05). Group differences for blood samples were also adjusted for age, sex, and season (dark season (months 10–12 and 1–3)/light season (months 4–9), and skin type (dark/light), using analysis of covariance (ANCOVA). For adjusted *p*-values see footnotes in the tables. Statistical analyses were performed with Statistica (version 12, StatSoft^®^, Tulsa, OK, USA), and *p*-values < 0.05 in the inference statistics were considered statistically significant.

## 3. Results

The basic characteristics of the study population are described in Table 1 as well as significant differences between controls and the other four patient groups (*p*-values), some of which were also adjusted for age, sex, season, and skin type (*p*-adjusted).

### 3.1. Anthropometric Variables

More women than men were included in all groups. Particularly notable was the gender difference in the group with fresh hip fractures, where three out of every four fracture patient was female.

All participants were over 75 years of age, but subjects in the healthy controls were younger than the other participants (mean age 77.9 years vs. 84.7; *p* < 0.001).

Patients living in nursing homes were oldest with a mean age of 86.4 years. There was no age difference between men and women within groups.

There was no difference in height between subjects in the groups among the male participants. Among women, however, healthy controls were taller than women in the other groups. BMI did not differ between groups.

Systolic and diastolic blood pressure were highest among the stroke subjects and lowest among the healthy controls and hip fracture patients.

### 3.2. Laboratory Findings

Patients in the group with hip fractures had, on average, lower plasma concentrations of calcium, albumin, and phosphorus levels than within the other groups. They had the highest plasma ALP concentrations as well.

Vitamin D status in the different groups are shown in Table 2.

Subjects in the control group had a mean 25(OH)D concentration of 74 nmol/L, which was higher than that of the stroke patients (64 nmol/L), the frequent emergency users (60 nmol/L), and the patients with hip fractures (51 nmol/L). The patients in the institutionalized homebound group had the lowest concentration of 25(OH)D (mean concentration 46.5 nmol/L); they also had the highest PTH and creatinine concentrations. Very few patients had measurable 25(OH) vitamin D2 concentrations. Only seven among the healthy controls, five stroke patients, and two of the emergency patients had 25(OH)D2 > 5 nmol/L. Therefore, S-25(OH)D concentrations depended mainly on the concentration of S-25(OH)D3.

S-25(OH)D showed a Gaussian distribution. Using the central 95% of the controls as a reference population for healthy older adults ≥ 75 years, the observed reference interval for S-25(OH)D for men and women was 31–123 nmol/L.

The mean 25(OH)D was highest in the control group 73.9 nmol/L and lowest in the institutionalized group (46.5 nmol/L). No gender differences were observed except for the patients in the frequent emergency care user group where females tended to have higher 25(OH)D values than males (*p* = 0.007).

### 3.3. Vitamin D Supplements and Other Prescribed Drugs

Vitamin D (cholecalciferol or calcitriol analogs) was prescribed to only a minority of patients (15.5%). These patients had significantly higher 25(OH)D levels (82.1 ± 2.4 vs. 49.0 ± 1.3 nmol/L, *p* < 0.001) and lower PTH (6.2 ± 0.6 vs. 8.1 ± 0.3 pmol/L, *p* = 0.003), than patients without vitamin D prescriptions. Serum 25(OH)D levels, prescribed D-vitamin dose, and D-vitamin status are shown in Table 2. Vitamin D status was slightly related to the number of prescribed drugs for continual use, all patients and controls combined. More drugs resulted in a lower 25(OH)D concentration (*p* = 0.02).

### 3.4. Seasonal Variation and Skin Type

**Figure 1:**
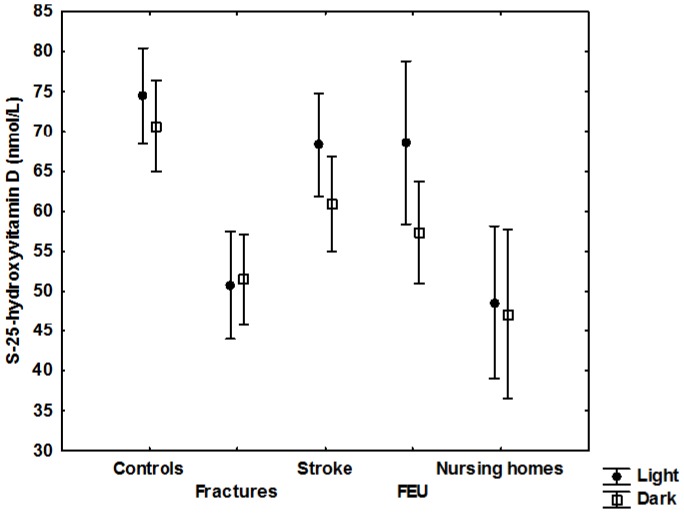
Serum-25-hydroxyvitamin D (nmol/L) levels due to seasonal variation (dark and light season of the year) including mean and 95% confidence interval. Adjusted for sex, age, and skin-type.

The season during which sample collection took place did not differ between groups, and 43% of the participants were examined during the summer months. There was no significant seasonal variation in mean 25(OH)D levels (Figure 1) in any of the groups. A tendency towards statistical significance was seen among the emergency patients (*p* = 0.058). When all patients were included, there was a small but significant seasonal variation when the light season (April to September) was compared with the dark season (October thru March); *p* = 0.03. Most patients and controls had Fitzpatrick skin type 2 or 3 (94%), whereas 4% had type 1, and 2% had type 4. No significant correlation between skin type and vitamin D status was seen.

## 4. Discussion

### 4.1. Vitamin D Levels in Older Adults

After 75 years of age, many individuals become increasingly frail, marked by serious mental and physical debilitation. Older adults are particularly at risk for developing vitamin D deficiency.

In this cross-sectional study, we sought to compare and investigate vitamin D levels in patients of the same age, but from different disease populations chosen from groups with a frailty phenotype, and from those with multiple comorbidities, whereby we suspected vitamin D deficiency to be disproportionally common in these groups.

We also examined a control group with older adults free from serious illness and who considered themselves completely healthy. All participants in the study were at least 75 years old. In line with their morbidity state, patients with acute hip fracture, stroke, institutionalized patients, and frequent users of emergency care demonstrated disproportionally low serum levels of 25(OH)D in comparison to healthy controls, who, on the other hand, had surprisingly high 25(OH)D concentrations. The mean serum 25(OH)D level of the healthy control group (74 nmol/L) was similar to a suggested optimal concentration based on physiological data and mortality studies and much higher than that of many officially recommended cut-off levels for vitamin D deficiency (<50 nmol/L). These results support the conclusion from other studies that a serum 25(OH)D level around 70–75 nmol/L is an optimal level for older adults.

### 4.2. Vitamin D Status in Study Patients with An Osteoporotic Hip Fracture

The first disease group was older adult patients with acute low energy hip fractures. Both old age and vitamin D deficiency are well-known risk factors for osteoporosis and hip fractures [15], and sustaining a hip fracture in old age is serious and associated with high morbidity and mortality. Classical clinical consequences of vitamin D deficiency include effects on proximal myopathy and myalgia, which can partly explain the increased risk of falling and fractures in older, vitamin D deficient, patients [16].

Vitamin D deficiency impairs bone mineralization and increases bone turnover via secondary hyperparathyroidism, thus accelerating bone loss and increasing fracture risk. In our study, PTH was significantly inversely correlated to 25(OH)D in all subjects, *p* < 0.001, but did not significantly differ from controls (*p* = 0.09).

Vitamin D, often taken in combination with calcium, is a common treatment for osteoporosis, and has also been shown to prevent falls in older adults [17,18]. Optimal benefits of vitamin D substitution have been observed at minimum doses of 700 to 1000 IU vitamin D per day, or a mean of 25(OH)D between 75 and 110 nmol/L [19]. In the current study, 23% of the hip fracture patients had vitamin D prescribed, but only 15% reached levels above 75 nmol/L, which is a suggested cut-off level by the International Osteoporosis Federation (IOF) [20], American Geriatric Society [21], and the Endocrine Society [22]. A majority of patients had levels below 50 nmol/L, which is the cut-off limit suggested for bone health by the US-American IOM report [23] and from the Nordic Nutrition Recommendations (NNR 2012) [13].

Sweden has a very high incidence of hip fractures [24], and the incidence has been shown to have both a seasonal and latitudinal variation, with the highest incidence in the north during the winter months. Variations in solar UVB doses and 25(OH)D concentrations have been proposed to explain this variation in hip fracture incidence [10]. In the current study, 25(OH)D levels did not differ significantly among hip fracture patients in the summer months compared with those in the winter months.

### 4.3. Vitamin D Status in Study Patients with Acute Stroke

The second group investigated included patients with acute stroke. Hypertension is a well-known risk factor for stroke, and there is also a well-known inverse correlation between 25(OH)D levels and blood pressure [25,26].

In addition to 25(OH)D levels, blood pressure and stroke are known to vary according to season and latitude [27,28]. In our study, we could not observe a statistical difference in S-25(OH)D between the winter months (dark season) compared with the summer months (light season) of the year (*p* = 0.19). The mean 25(OH)D levels in stroke patients were higher than among hip fracture patients, but significantly lower than in the healthy controls.

The finding of relatively high levels of 25(OH)D in stroke patients (mean 64 ± 27 nmol/L) was somewhat surprising. Several studies, including several meta-analyses of multiple prospective cohort studies, have shown an increased risk for stroke in people with low vitamin D levels [29,30,31,32,33].

Most of the stroke patients had 25(OH)D levels above 50 nmol/L, thus, vitamin D does not appear to have a clear stroke preventive effect in an older Swedish population. The reason for the relatively high levels of 25(OH)D in the stroke population of this study is unclear, but, interestingly, Wang and co-workers found an inverse association between circulating 25(OH)-vitamin D in the range of 20–60 nmol/L and the risk for CVD, but a higher risk for stroke when 25(OH)D was higher than 60 nmol/L [31].

### 4.4. Vitamin D Status in Study Patients with ≥4 Emergency Care Visits per Year

Apart from the well-known effects of vitamin D on bone health, calcium, and phosphate metabolism, in recent years, the focus of research has been on the so-called non-classical effects of vitamin D. Vitamin D is a potent regulator of many important physiological responses, and low vitamin D levels have been associated with many chronic and acute diseases, such as insulin resistance, cardiovascular disease, cancer, autoimmune diseases, psychiatric illness, and infections. Results from randomized controlled trials are, however, conflicting, and low 25(OH)D has been suggested only as a marker of ill health for many of these conditions. Recent studies have also suggested that vitamin D could be a marker of resistance to fatality of potentially fatal diseases, in other words, sufficient vitamin D status may be needed to regulate responses by the immune system, when challenged by severe diseases, to prevent fatality [34].

As a third group we, therefore, chose to investigate a group of older adults defined as frequent users of emergency care. These people frequently pose special diagnostic challenges and often have nonspecific, vague complaints, and atypical presentations of a variety of diseases. The patients in our study used emergency care at the local hospital four or more times a year. Also, this group of patients had significantly lower 25(OH)D levels compared with that of controls.

### 4.5. Vitamin D Status in Study Patients Living in Nursing Homes

Elderly, multi-ill persons, living in nursing homes, have poor access to outdoor activities and may develop vitamin D deficiency. As a fourth group we, therefore, included a group of homebound, community-dwelling elderly persons in the study. These patients had very low 25(OH)D concentrations, and 75% had levels lower than 50 nmol/L. Only 14% had vitamin D prescribed. This is not surprising, since vitamin D deficiency is a frequent finding among older adults in most countries, regardless of latitude, and especially in institutionalized older adults. This is an important finding whereby vitamin D deficiency in these patients has been associated with increased mortality [3]. We observed no seasonal variations in this group of patients. Mean age for this group was 86. Aging decreases the capacity of human skin to produce vitamin D3. Many institutionalized older adults spend little time outdoors, which could explain the lack of seasonal variation for both this group and the hip fracture group.

### 4.6. Vitamin D Status in Healthy Controls

Surprisingly, we detected no seasonal variation in the control group. This is in contrast to recent findings in healthy, Swedish blood donors (mean age 41 ± 13 years) where S-25(OH)D varied greatly over the year, correlating with the intensity of UVB irradiation [9]. Our much older control subjects had a mean 25(OH)D concentration of 73.9 nmol/L, and levels did not, as was expected, differ significantly between summer and winter. No one in the control group had vitamin D prescribed, but we had no information regarding intake of food sources rich in vitamin D (cholecalciferol or ergocalciferol) or vitamins not prescribed by a physician. Nor did we obtain information on outdoor exposure to sunlight, or vacations abroad, etcetera.

On the other hand, in a prospective study from southern Sweden [15], no significant seasonal variation was seen in 25(OH)D values (ranging from 58 to 66 nmol/L) as well as in another Swedish prospective study investigating older healthy Swedish women aged 75 and above [35]. It, thus, seems that seasonal variations in vitamin D status are less pronounced in very old, healthy individuals compared to that of younger persons. We speculate that our control group, consisting of retired, but healthier and more active individuals, may travel to southerly latitudes more than would otherwise be expected for this age group, thus gaining greater exposure to sunlight, and consequently higher levels of vitamin D. Another explanation is that these healthy older adults have different eating habits with a higher intake of vitamin D, and that in older individuals, the dietary importance of vitamin D levels are more significant, compared to that of younger individuals, while sunlight’s role is less important. There could also have been more individuals in this healthy group with a genetic predisposition for higher blood levels of 25(OH)D over the year.

### 4.7. Current Guidelines for Optimal Vitamin D Status

Interestingly, the mean vitamin D concentrations we observed in the healthy control group (74 nmol/L) was much higher than that of the Swedish recommended cut-off level for vitamin D deficiency (>50 nmol/L).

There is a relative consensus throughout the world that serum levels below 25 nmol/L (10 ng/ml) qualify as ‘deficient’, but there is some controversy regarding normal values. It is also important to note that “normal” vitamin D status using data from older studies may be difficult to assess since serum 25(OH)D measurements have proven to be a major challenge, with considerable variation in results between laboratories and methods used in earlier studies [36,37].

The frequently referenced report from the American Institute of Medicine (IOM) from 2011 recommended that a serum 25(OH)D level of at least 50 nmol/L would be sufficient to optimize bone health. The same cut-off level was recommended in the Nordic Nutrition recommendation, released in October 2013. In contrast to the USA’s IOM report, other reports and guidelines, such as from the American Endocrine Society (2010), the International Osteoporosis Foundation (2010), and the American Geriatric Society (2014), have suggested that a minimum level of 75 nmol/L is necessary in older adults. This is based on the assumption that effective serum 25(OH)D levels are lower for skeletal disease than for non-skeletal disease and premature mortality, and that higher levels are needed to minimize, for example, the risk of falls and increase life span among frail, older adult subjects [38].

In the recent Danish, CopD-study, serum 25(OH)D levels were analyzed from 247,574 subjects from the Copenhagen general practice sector [39]. In that study, a serum level of approximately 70 nmol/L was associated with the lowest total mortality risk. Lower levels, but also higher levels in this study, were associated with increased risk. Interestingly, and despite their advanced age, controls in our study had, on average, nearly the same 25(OH)D levels (74 nmol/L) as the optimal level in the CopD-study. This level has also been suggested as the optimal level based on physiological data. Adequate dietary calcium absorption depends on levels of 25(OH)D, and maximum absorption of dietary calcium occurs at a 25(OH)D level of approximately 70 nmol/L or higher. Since blood calcium levels must be maintained, vitamin D deficiency leads to secondary hyperparathyroidism and decreased bone mineralization [40].

Self-rated health provides a good indication of health, and has been proven to be a predictor of mortality [41,42]. Since the subjects in our control group considered themselves to be in excellent health, and since their mean serum level of 25(OH)D was 74 nmol/L, our study supports the conclusion from other studies that, in terms of mortality, 70–75 nmol/L is an optimal level for 25(OH)D. However, due to the cross-sectional design of the current study, it is not possible to infer causality between 25(OH)D levels and health outcomes, underlining the need for further randomized trials with larger populations. On the whole, most of these trials have not demonstrated improved health in vitamin D-treated individuals, with the exception of life expectancy for older adults.

### 4.8. Limitations in the Study

The present study was, as noted, limited by its cross-sectional design.

A single measurement may not accurately reflect the relationship between 25(OH)D and health outcomes over time [15]. A validated food frequency questionnaire for assessing D-vitamin and calcium intake, as well as collecting information on lifestyle factors such as sun habits and travelling abroad, could have been helpful for understanding the vitamin D status in our population. A further shortcoming was the lack of questions to the control group regarding their exposure to sunlight, and an additional limitation was the slightly lower mean age of the control group compared to the other groups.

## 5. Conclusions

In the current study, we showed that vitamin D status was low in Swedish people aged 75 or older with high disease burden. In contrast, high self-perceived health in older adults was associated with adequate serum levels of 25(OH)D. These results support the conclusion from other studies that a serum 25(OH)D level around 70–75 nmol/L is an optimal level for older adults.

## Figures and Tables

**Table 1:** Anthropometric variables and laboratory findings of the five groups in the study population: healthy controls, stroke patients, orthopedic patients with fragility fractures, frequent users of emergency departments (FEU), and homebound older persons living in nursing homes (Nursing homes).

	Controls	Fractures	Stroke	FEU	Nursing Homes
*N*	169	133	122	81	51
**Gender**					
Males (*n*; %)	67 (40)	33 (25)	52 (43)	37 (46)	25 (50)
Females (*n*; %)	102 (60)	100 (75)	70 (57)	44 (54)	25 (50)
**Age (Years)**					
**Males**					
Mean (SD)	77.9 (4.0)	84.4 (5.4)	82.4 (4.9)	82.2 (4.6)	86.0 (5.4)
Median (Range)	76 (75–90)	85 (76–94)	83 (75–95)	82 (75–91)	86 (76–96)
*p*-value vs. ctrls	-	<0.001	<0.001	<0.001	<0.001
**Females**					
Mean (SD)	78.0 (3.1)	86.1 (5.5)	84.7 (5.5)	84.7 (5.6)	86.4 (5.1)
Median (Range)	77 (75–87)	86 (75–98)	85 (75–97)	85 (75–94)	86 (75–99)
*p*-value vs. ctrls	-	<0.001	<0.001	<0.001	<0.001
**Height (cm)**					
**Males**					
Mean (SD)	177 (6)	175 (8)	175 (6)	175 (6)	176 (10)
Median (Range)	176 (165–194)	174 (161–196)	174 (162–192)	175 (163–187)	176 (144–194)
*p*-value vs. ctrls	-	ns	ns	ns	ns
**Females**					
Mean (SD)	163 (5)	160 (7)	160 (6)	162 (7)	160 (5)
Median (Range)	163 (152–175)	160 (145–188)	161 (140–173)	160 (145–173)	160 (150–168)
*p*-value vs. ctrls	-	0.008	0.033	<0.001	0.020
**BMI (kg/m^2^)**					
Mean (SD)	24.9 (3.5)	24.7 (4.6)	25.3 (4.6)	25.5 (4.6)	25.9 (4.5)
Median (Range)	24.7 (16.0–41.6)	24.4 (15.4–39.4)	25.0 (15.2–39.3)	24.9 (15.9–38.6)	25.8 (17.1–38.3)
*p*-value vs. ctrls	-	ns	ns	ns	ns
**Systolic blood pressure (mmHg)**					
Mean (SD)	135.3 (14.4)	133.8 (17.7)	154.0 (22.1)	142.4 (21.7)	141.8 (20.9)
Median (Range)	140 (93–165)	135 (100–190)	154 (95–220)	140 (95–195)	140 (90–200)
*p*-value vs. ctrls	-	0.136	<0.001	0.022	0.092
**Diastolic blood pressure (mmHg)**					
Mean (SD)	70.6 (9.0)	68.7 (8.7)	77.6 (13.2)	71.3 (11.8)	76.7 (12.2)
Median (Range)	70 (50–100)	70 (50–90)	80 (40–110)	70 (50–95)	75 (55–105)
*p*-value vs. ctrls	-	0.038	<0.001	0.792	0.004
**Serum ionized calcium (mmol/L)**					
Mean (SD)	1.25 (0.04)	1.22 (0.07)	1.26 (0.05)	1.26 (0.13)	1.26 (0.06)
Median (Range)	1.25 (1.15–1.41)	1.22 (1.06–1.55)	1.25 (1.15–1.48)	1.25 (1.09–2.26)	1.26 (1.17–1.50)
*p*-value vs. ctrls	-	<0.001	0.646	0.823	0.337
*p*-values adjusted *		0.014	0.605	0.343	0.342
**Plasma calcium (mmol/L)**					
Mean (SD)	2.3 (0.1)	2.2 (0.1)	2.3 (0.1)	2.3 (0.1)	2.3 (0.1)
Median (Range)	2.3 (2.1–2.6)	2.2 (1.9–2.7)	2.3 (2.1–2.7)	2.3 (1.9–2.7)	2.3 (2.1–2.6)
*p*-value vs. ctrls	-	<0.001	0.589	0.120	0.514
*p*-values adjusted *	-	<0.001	0.479	0.177	0.784
**Plasma albumin (mmol/L)**					
Mean (SD)	42.5 (2.3)	31.1 (3.5)	40.1 (4.1)	37.5 (4.4)	39.8 (3.1)
Median (Range)	42 (36–48)	31 (23–41)	40 (32–51)	38 (23–49)	40 (32–46)
*p*-value vs. ctrls	-	<0.001	<0.001	<0.001	<0.001
*p*-values adjusted *	-	<0.001	<0.001	<0.001	<0.001
**Plasma phosphorus (mmol/L)**					
Mean (SD)	1.22 (0.15)	1.15 (0.24)	1.24 (0.20)	1.17 (0.21)	1.25 (0.16)
Median (Range)	1.2 (0.8–1.6)	1.1 (0.6–2.2)	1.2 (0.8–1.7)	1.2 (0.7–1.9)	1.3 (0.8–1.7)
*p*-value vs. ctrls	-	<0.001	0.894	0.009	0.412
*p*-values adjusted *	-	0.008	0.663	0.046	0.442
**Serum Parathyroid hormone (pmol/L)**					
Mean (SD)	5.84 (1.67)	7.27 (4.69)	7.26 (3.86)	7.90 (4.48)	9.50 (8.97)
Median (Range)	5.6 (3.1–11.0)	5.9 (1.9–36.0)	6.5 (1.9–20.0)	6.8 (1.9–25.0)	7.1 (3.1–57.0)
*p*-value vs. ctrls	-	0.103	0.009	0.001	<0.001
*p*-values adjusted *	-	0.094	0.052	0.005	<0.001
**Plasma Alkaline phosphatase (µkat/L)**					
Mean (SD)	1.12 (0.27)	1.72 (0.82)	1.32 (0.49)	1.59 (1.25)	1.41 (0.47)
Median (Range)	1.1 (0.6–1.8)	1.5 (0.6–6.1)	1.2 (0.7–4.1)	1.3 (0.6–11.0)	1.3 (0.4–3.2)
*p*-value vs. ctrls	-	<0.001	<0.001	<0.001	<0.001
*p*-values adjusted *	-	<0.001	0.003	<0.001	<0.001
**B-HbA1c (mmol/mol)**					
Mean (SD)	38.1 (3.9)	39.6 (9.5)	43.6 (10.6)	43.6 (12.4)	44.2 (13.7)
Median (Range)	38 (30–59)	38 (28–119)	40 (31–103)	40 (32–93)	39 (29–85)
*p*-value vs. ctrls	-	0.620	<0.001	<0.001	0.033
**Plasma creatinine (mmol/L)**					
Mean (SD)	77.8 (15.6)	95.2 (52.4)	91.1 (32.5)	104.6 (55.0)	103.4 (35.4)
Median (Range)	76 (46–135)	81 (40–405)	83 (46–255)	88 (39–450)	96 (53–211)
*p*-value vs. ctrls	-	0.040	0.001	<0.001	<0.001

*p*-values from Mann-Whitneys U-test, provided Kruskal-Wallis nonparametric ANOVA was statistically significant (*p* < 0.05). For adjusted *p*-values see Footnotes; * Adjusted for age, sex, season and skin (dark/light) using analysis of covariance (ANCOVA). Log transformation was made for PTH and ALP.

**Table 2:** Vitamin D status, vitamin D supplements, and number of other prescribed drugs of the five groups in the study population.

	Controls	Fractures	Stroke	FEU	Nursing Homes
*N*	169	133	122	81	51
**Serum-25-hydroxyvitamin D (nmol/L)**					
**Mean (SD)**	73.9 (22.3)	50.8 (21.8)	63.8 (27.1)	60.0 (27.6)	46.5 (26.7)
**Median (Range)**	74 (22–154)	50 (10–128)	62 (16–135)	56 (16–165)	38 (15–132)
***p*-value vs. ctrls**	-	<0.001	<0.001	<0.001	<0.001
*p*-values adjusted *	-	<0.001	0.005	<0.001	<0.001
**Serum-25-hydroxyvitamin D (nmol/L) no supplements**					
**Mean (SD)**	73.9 (22.3)	44.6 (16.6)	56.7 (23.1)	51.1 (22.0)	39.7 (19.5)
**Median (Range)**	74 (22–154)	44 (10–84)	55 (16–135)	48 (16–109)	35 (15–107)
***p*-value vs. ctrls**	-	<0.001	<0.001	<0.001	<0.001
*p*-values adjusted *	-	<0.001	<0.001	<0.001	<0.001
**Serum-25-hydroxyvitamin D (nmol/L; *n*; %)**					
**<25**	2 (1.2)	11 (8.3)	10 (8.2)	6 (7.4)	6 (11.8)
**25-49**	20 (12)	55 (41)	28 (23)	26 (32)	32 (63)
**50-74**	65 (38)	47 (35)	45 (37)	27 (33)	4 (8)
**≥75**	82 (49)	20 (15)	39 (32)	22 (27)	8 (16)
**Serum-25-hydroxyvitamin D (nmol/L; *n*; %) no supplements**					
**<25**	2 (1.2)	10 (9.7)	9 (9.5)	6 (10.2)	6 (13.6)
**25-49**	20 (12)	52 (50)	26 (27)	24 (41)	32 (73)
**50-74**	65 (38)	37 (36)	41 (43)	21 (36)	3 (7)
**≥75**	82 (49)	4 (4)	19 (20)	8 (14)	3 (7)
**D-vitamin supplements prescribed (*n*; %)**					
**No**	169 (100)	103 (77)	95 (78)	59 (73)	44 (86)
**Yes**	0 (0)	30 (23)	27 (22)	22 (27)	7 (14)
**D-vitamin daily dose (prescribed)**					
**No**	169 (100)	103 (77)	95 (78)	59 (73)	43 (84)
**400 IU**	0 (0)	7 (5)	5 (4)	6 (7)	3 (6)
**800 IU**	0 (0)	20 (15)	21 (17)	14 (17)	3 (6)
**>800 IU**	0 (0)	1 (1)	1 (1)	0 (0)	0 (0)
**Calcitriol**	0 (0)	2 (2)	0 (0)	2 (2)	0 (0)
**Pharmacy**					
**Number of prescribed drugs**					
**Mean (SD)**	2.3 (2.6)	5.8 (3.2)	5.4 (3.1)	7.8 (3.3)	6.9 (3.8)
**Median (Range)**	2 (0–10)	5 (0–14)	5 (0–12)	8 (1–17)	7 (0–14)
***p*-value vs. ctrls**	-	<0.001	<0.001	<0.001	<0.001

*p*-values from Mann-Whitneys U-test, if Kruskal-Wallis nonparametric ANOVA was statistically significant (*p* < 0.05). For adjusted serum-values see footnotes; * Adjusted for age, sex, season (summer/winter) and skin (dark/light) using analysis of covariance (ANCOVA).
